# Polymorphous Low Grade Adenocarcinoma of the Parotid in a Teenager

**Published:** 2017-09

**Authors:** Divya Khosla, Shalini Verma, Nitin Gupta, Rajpal-S Punia, Gurbir Kaur, Awadhesh-K Pandey, Kislay Dimri

**Affiliations:** 1 *Department of Radiotherapy, Government Medical College and Hospital, Chandigarh, India.*; 2 *Department of Otorhinolaryngology and Head and Neck Surgery, Government Medical College and Hospital, Chandigarh, India.*; 3 *Department of Pathology, Government Medical College and Hospital, Chandigarh, India.*

**Keywords:** Adenocarcinoma, Parotid gland, Salivary gland neoplasms, Surgery

## Abstract

**Introduction:**

Polymorphous low grade adenocarcinoma (PLGA) is a rare salivary gland neoplasm with an indolent course. It occurs primarily in the minor salivary glands but can rarely occur in the major salivary glands. It usually occurs in the fifth to seventh decades of life with female preponderance.

**Case Report:**

A 16-year-old male presented with recurrent painless swelling in the right preauricular region and with a history of surgical intervention at the same site in the past. His histopathology report was suggestive of pleomorphic adenoma. The swelling recurred after one year of excision and a superficial parotidectomy was performed. The detailed histopathological examination was suggestive of Polymorphous low grade adenocarcinoma. In view of close margins, the patient was given adjuvant radiotherapy. Thirty-three months post-surgery, he is alive and disease-free. We describe a rare case of PLGA of the parotid gland in a teenager with its clinical characteristics, histopathological features, and treatment.

**Conclusion:**

The occurrence of PLGA in the parotid gland is rare with only a few cases reported in literature. The diagnosis of PLGA is challenging due to morphological diversity.

## Introduction

Polymorphous low grade adenocarcinoma (PLGA) was described as terminal duct carcinoma by Batsakis et al (1) and lobular carcinoma by Freedman and Lumerman in 1983 (2). Evans and Batsakis (3), in their study of 14 cases, designated it as PLGA describing its low grade behaviour and diverse morphology. The diagnosis of PLGA is often difficult due to its morphological diversity with variable growth patterns. PLGA occurs most commonly in minor salivary glands of the oral cavity and oropharynx. The most frequent site of presentation is the palate, accounting for about 60% of all PLGAs, followed by lip, buccal mucosa, and alveolar ridge. It may also affect the retromolar region, floor of mouth, posterior tongue and nasal cavity (4-6). PLGA occurs rarely in major salivary glands and can arise de novo or in a pre-existing pleomorphic adenoma (7-9). It occurs over a wide age range with the majority of cases occurring in the fifth to seventh decade of life and rarely in the first and second decade (5). PLGAs originating from major salivary glands have clinical and pathological characteristics similar to those arising from minor salivary glands (7). Herein, we report a rare case of PLGA arising from the parotid gland in a teenager with its clinicopathological features and review of literature.

## Case Report

A 16-year-old-boy came to our clinic with a 3-year history of painless swelling in the preauricular region. He underwent surgical excision one year ago and the histopathological examination was suggestive of pleomorphic adenoma. The complete details were not available as it was performed in another institution. The swelling recurred one year post excision at the same site. 

**Fig 1:**
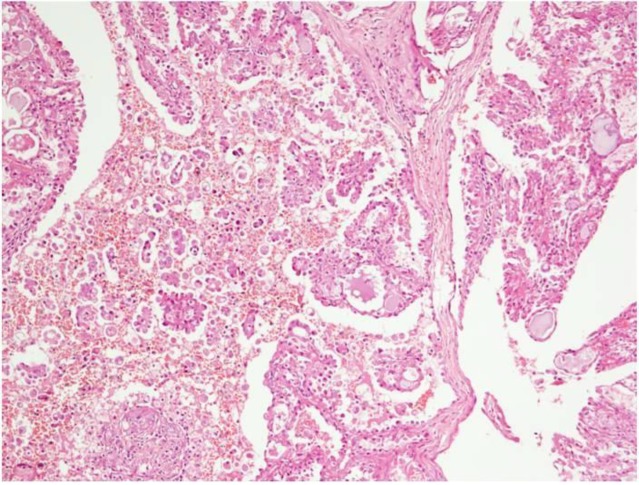
Lobular nests admixed with cribriform and focal papillary cystic areas and ductal elements (H&E x 200

During the examination, a firm swelling of 3 cm x 3 cm was palpable in the right preauricular region. Haematological, biochemical renal and liver function tests were within normal limits. Chest X-ray and ultrasound of abdomen and pelvis were normal. Contrast enhanced computed tomography (CECT) was suggestive of a bulky right parotid with iso to hyperdense lesion in the superficial lobe. Fine needle aspiration cytology from the parotid swelling was suggestive of pleomorphic adenoma. The patient underwent right superficial parotidectomy. Gross examination of the specimen revealed a grey white infiltrating tumor measuring 2.5 cm x 2 cm x 2 cm with focal grey brown areas. Microscopically, a diffusely infiltrating tumor in the salivary parenchyma and adjoining fibroadipose tissue was observed. This tumor was arranged in lobules, papillary-cystic, tubular and focally in cribriform pattern (Fig.1).

**Fig 2:**
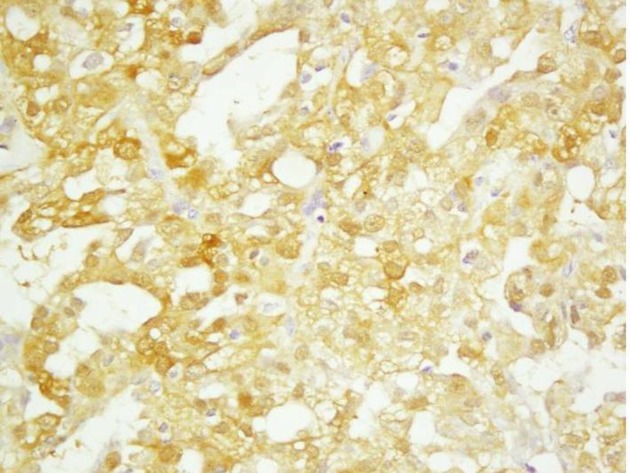
Polymorphous low grade adenocarcinoma showing diffuse staining pattern for S-100

The tumor cells showed mild anaplasia with vesicular chromatin, pin-point nucleoli clear to eosinophilic cytoplasm, and few mucin filled cystic spaces. There were no areas of atypical mitosis or necrosis. Intervening areas showed fibrosis and mild lymphomononuclear cell infiltrate. The tumor cells showed positivity for S-100 (Fig.2). 

The patient received postoperative radiotherapy in view of close margins for a total dose of 50.4 Gy in 28 fractions. The patient is doing well 33 months post surgery without any clinical or radiological evidence of recurrence.

## Discussion

PLGA is a low-grade neoplasm of minor salivary glands with an indolent nature, which usually presents as a painless slow growing mass. It mainly affects female patients and the female to male ratio is 2:1 (5,6). It occurs over a wide age range (16-94 years), with the mean age of presentation at 59 years. It has also rarely been reported in adolescents (5,6,10-13). PLGA in major salivary glands is a rare occurrence. Lymphatic and haematogenous metastasis are uncommon. 

PLGA is characterized by a triad of infiltrative growth pattern, cytological uniformity and architectural diversity (14). Grossly it appears as a firm, well circumscribed, non-encapsulated, yellowish lobulated mass that invades adjacent tissues. The most characteristic feature of PLGA is the morphological diversity existing between tumors and within a tumor (10). The various growth patterns include solid, lobular, trabecular, glandular, micro-cystic, cribriform, fascicular, tubular, and papillary. Mitosis and necrosis are infrequent but perineural invasion is frequently seen (14). PLGA poses diagnostic challenges due to its morphological variations and is often mistaken as pleomorphic adenoma and adenoid cystic carcinoma (8,14). The presence of infiltrative margins, neurotropism and absence of chondromyxoid stroma differentiates PLGA from pleomorphic adenoma. Pleomorphic adenomas are usually well circumscribed and are composed of bilayered ductal or gland like structures surrounded by myoepithelial cells. Immunohistochemical staining with glial fibrillary acidic protein may be helpful in differentiating pleomorphic adenoma from PLGA (14). The differentiation between PLGA and adenoid cystic carcinoma is based primarily on cytological features. Perineural invasion is a feature of both PLGA and adenoid cystic carcinoma but the presence of targetoid arrangement of perineural invasion is characteristic of PLGA. Presence of calcific deposits, papillary growth and lack of nuclear pleomorphism in solid areas favours PLGA. The presence of hyperchromatic and angulated nuclei also helps in distinguishing adenoid cystic carcinoma from PLGA on biopsy samples (10,14).

Complete surgical excision with adequate margins is the treatment of choice for PLGA. Recurrence may occur after years, hence long term follow-up is recommended. Vincent et al. (4) reported a recurrence rate of 17% with a regional metastasis rate of 9%. Nearly one-third of patients experienced recurrence in a series of 40 patients by Evans et al (6). Due to the rarity of this entity, the role of adjuvant radiotherapy is still unclear. Our patient was given adjuvant radiotherapy in view of close margins. 

## Conclusion

PLGA arising from the parotid gland is a rare occurrence. The diagnosis of PLGA is difficult and challenging due to its morphological diversity. Wide local excision with negative margins is the treatment of choice. The role of adjuvant radiotherapy is still not defined due to the rarity of this disease and limited literature. The present case is unique because of the younger age of presentation and rare location.
